# Animal models for human herpesvirus 6 infection

**DOI:** 10.3389/fmicb.2013.00174

**Published:** 2013-07-04

**Authors:** Joséphine M. Reynaud, Branka Horvat

**Affiliations:** International Center for Infectiology Research, INSERM U1111, CNRS UMR5308, ENS Lyon, University of Lyon 1Lyon, France

**Keywords:** HHV-6, animal model, mouse, monkey, HIV, AIDS, neuroinflammation, CD46

## Abstract

Human herpesvirus (HHV)-6A and HHV-6B are two enveloped DNA viruses of β-herpesvirus family, infecting over 90% of the population and associated with several diseases, including *exanthema subitum* (for HHV-6B), multiple sclerosis and encephalitis, particularly in immunosuppressed patients. Animal models are highly important to better understand the pathogenesis of viral infections. Naturally developed neutralizing antibodies to HHV-6 or a related virus were found in different species of monkeys, suggesting their susceptibility to HHV-6 infection. Both HHV-6 DNA and infectious virus were detected in experimentally infected Cynomolgus and African green monkeys, although most animals remained clinically asymptomatic. Furthermore, HHV-6A infection was shown to accelerate the progression of AIDS (acquired immunodeficiency syndrome) in macaques and to lead to the development of neurological symptoms in the marmoset model. Humanized SCID (severe combined immunodeficiency) mice efficiently replicated HHV-6 and were also susceptible to coinfection with HHV-6 and HIV-1 (human immunodeficiency virus 1). As CD46 was identified as a receptor for HHV-6, transgenic mice expressing human CD46 may present a potentially interesting model for study certain aspects of HHV-6 infection and neuroinflammation.

## INTRODUCTION

Human herpesvirus (HHV)-6 belongs to the β-Herpesviridae subfamily, together with its closest homolog HHV-7 and human cytomegalovirus (HCMV). The two variants of HHV-6, HHV-6A and HHV-6B, have recently been recognized as two distinct viruses by the international committee on taxonomy of viruses, based mostly on their known genetic and epidemiological features (Braun et al., 1997). Primary infection with HHV-6B was identified as the etiological cause for roseola (*exanthema subitum*), a common febrile illness in infants (Yamanishi et al., 1988), whereas primary infection with HHV-6A has not yet been clearly associated to any specific disease. Like most herpesviruses, HHV-6A and -6B are able to establish asymptomatic long-term persistence in their hosts, and can reactivate under specific conditions. Although the mechanisms of reactivation are not yet completely understood, both viruses are known to reactivate in immunosuppressed patients, causing a variety of complications such as encephalitis, hepatitis, or graft rejection (Dockrell and Paya, 2001; Zerr, 2006). In addition, HHV-6A and -6B have been associated with several neurological diseases in the immunocompetent population. Indeed, numerous clinical studies have established a correlation between HHV-6A and -6B infection and the demyelinating, autoimmune disease-multiple sclerosis (reviewed in Reynaud and Horvat, 2013), and both viruses are thought to be involved in the development of certain cases of encephalitis, meningitis, and epilepsy (Theodore et al., 2008; Yao et al., 2010).

Human herpesvirus-6 has often been isolated from patients with acquired immunodeficiency syndrome (AIDS) and was suggested to play a role in the progression of this disease. Indeed, an active and wide-spread HHV-6 infection was observed in AIDS patients (Knox and Carrigan, 1994; Secchiero et al., 1995) and AIDS was described to progress rapidly after primary HHV-6 infection in children with vertically inherited human immunodeficiency virus (HIV; Kositanont et al., 1999). Both HHV-6 and HIV have a preferential tropism toward CD4^+^ T cells and can establish simultaneous productive infection with synergistic cytopathic effects (Lusso et al., 1989). Moreover, HHV-6 has a wider range of susceptible cell types than HIV-1 and was shown to induce the expression of the HIV-1 receptor CD4 on immune cells that do not naturally express this molecule, rendering them, thus, susceptible to HIV-1 infection (Lusso et al., 1993, 1995). However, in the context of both AIDS and different other HHV-6-related pathologies, the consequences of coinfection and the potential mechanisms involved in the pathogenesis remain to be elucidated.

A few antiviral drugs have been shown to be efficient against HHV-6 infection *in vitro *(Manichanh et al., 2000; De Clercq et al., 2001; De Bolle et al., 2005b) and were successfully used for the treatment of patients suffering from encephalitis following viral reactivation (reviewed in De Bolle et al., 2005b). However, these treatments are often associated with strong adverse effects and fully controlled specific clinical studies demonstrating their *in vivo* efficiency are still missing. Animal models represent very useful tools for preclinical analyses of potential antiviral drugs and for the study of viral pathogenesis. Here, we review the different animal models developed for the study of HHV-6A and/or HHV-6B infection (**Table 1**) and discuss the data obtained. In particular, the use of animal models has brought new evidence of the capacity of HHV-6A to induce neuropathology and has allowed the study of the interactions between HHV-6 and immunodeficiency viruses, showing a role of HHV-6A in AIDS progression and providing potential explanations for the impact of HHV-6A on the course of HIV infection.

**Table 1:** Described animal models for HHV-6 infection.

Species	Genetic modification	Virus (strain)	Route of inoculation	Clinical signs	Virological data	Reference
African green monkey (*Cercopithecus aethiops*)	none	HHV-6B (HST)	s.c./i.v	Skin rash (1 animal)	IgG response, viral DNA (PBMC, l.n.)	Yalcin et al. (1992)
Cynomolgus macaque (*Macaca fascicularis*)	none	HHV-6B (HST)	s.c./i.v.	none	IgG response, viral DNA (PBMC, spleen)	Yalcin et al. (1992)
Pig-tailed macaque (*Macaca nemestrina*)	none	HHV-6A (GS)	i.v.	fever, nasal discharge, splenomegaly, lymphadenopathy, abdominal rash	IgG response, plasma viremia, viral transcripts (l.n.)	Lusso et al. (2007)
Marmoset (*Callithrix jacchus*)	none	HHV-6A (U1102)	i.v.^*^	motor weaknesses, sensorial abnormalities, facial palsy, lesions in the corpus callosum at MRI	IgG and IgM response, viral DNA (brain, spleen, l.n., heart, kidney, liver)	Leibovitch et al. (2013)
	none	HHV-6B (Z29)	i.v.^*^	none	IgG response, viral DNA (brain)	Leibovitch et al. (2013)
	none	HHV-6A (U1102)	i.n.^*^	none	Plasma viremia, viral DNA (saliva, PBMC)	Leibovitch et al. (2013)
Mouse* (Mus musculus)*	none	HHV-6B (Z29)	i.p.	none	IgG response	Svensson et al. (2010)
	none	HHV-6A	N/A	none	N/A	Lusso (1996)
	Hu SCID (Thy/liv)	HHV-6A (GS) HHV-6B (PL-1)	in the implant	N/A	N/A	Gobbi et al. (1999)

* Inoculation performed using several (three or four) injections, s.c., subcutaneous; i.v., intravenous; i.n., intanasal; i.p., intraperitoneal; l.n., lymph nodes; N/A, not available.

## SIMIAN MODELS

### NATURAL INFECTION IN MONKEYS

Shortly after the discovery of HHV-6, several groups have searched for evidence of natural infection by HHV-6 in monkeys. Initial studies first reported an absence of specific antibodies in several species of new- and old-world non-human primates (Salahuddin et al., 1986; Lusso et al., 1990). In contrast, another study carried out on 10 different species of monkeys revealed the presence of HHV-6-reactive antibodies, suggesting a previous infection either by HHV-6 or a closely related virus (Higashi et al., 1989). Among the tested species, eight were positive in immunofluorescence assay and seroneutralization. African green monkeys, squirrel monkeys, chimpanzees, and orangutan appeared to be the most frequently infected, with 75–100% of prevalence. Furthermore, several groups of monkeys of the same species but from different locations exhibited similar prevalence rates, thus suggesting that the susceptibility to HHV-6 infection may be species-dependent.

More recently, a simian homolog of HHV-6 was isolated from blood samples from chimpanzees (*Pan troglodytes*; Lacoste et al., 2005). This new member of the β-herpesvirus group, called PanHV6, was found to be particularly close to the Z29 strain of HHV-6B. It was detected in several different subspecies of wild-caught chimpanzees from Cameroon and Gabon, but also in animals born in captivity in the Netherlands, indicating that this virus is present in different populations of chimpanzees. Several simian homologs of other human herpesviruses, including HCMV and Epstein–Barr virus (EBV; Davison et al., 2003; Ehlers et al., 2003) have been identified, which supports the theory that these viruses might have co-evolved with their host species. The natural susceptibility of some species of monkeys to infection with HHV-6 or a simian counterpart indicates that monkeys may represent an appropriate model for the study of HHV-6A and/or -6B pathogenesis.

### EXPERIMENTAL INFECTION IN SIMIAN MODELS

#### Infection of simian cells

Analyses performed on *in vitro*-infected peripheral blood mononuclear cells (PBMCs) from several species of monkeys, indicated that cells from chimpanzees and macaques (*Macaca nemestrina* and *M. mulatta*) are the most susceptible to infection by HHV-6 (Lusso et al., 1990, 1994). The infection of PBMCs led to the production of viral proteins and viral particles, observed by immunofluorescence and electron microscopy. Infection seemed highly variable among the species of monkeys tested and depended on the virus used (A or B). PBMCs from rhesus macaques (*M. mulatta*) were found to be susceptible to HHV-6B infection only, while PBMCs from pig-tailed macaques (*M. nemestrina*) were infected with similar efficiency by both HHV-6A and -6B.

#### African green monkeys and cynomolgus macaques

The first experiments of *in vivo *HHV-6 infection in monkeys were conducted using African green monkeys (*Cercopithecus aethiops*) and cynomolgus macaques (*M. fascicularis*; Yalcin et al., 1992). Four animals from each species were inoculated with the HST strain of HHV-6B. Monkeys received a single subcutaneous (s.c.) or intravenous (i.v.) injection of 10^5^ half maximal tissue culture infective doses (TCID_50_), and were monitored for 33 days. Following virus inoculation, a specific antibody response was detected, as well as the presence of viral DNA in the PBMCs and in the spleen and lymph nodes of some animals. However, the infection remained asymptomatic in all animals, except for one African green monkey, which developed a skin rash on the trunk (**Table 1**).

#### Pig-tailed macaques

Infection with HHV-6A was later described in pig-tailed macaques (*M. nemestrina*; Lusso et al., 2007). After i.v. inoculation with HHV-6A (GS strain), clinical symptoms of mild to moderate intensity were observed, including fever, nasal discharge, splenomegaly, generalized lymphadenopathy and abdominal rash (in one animal). Moreover, in this model, systemic infection was confirmed by the detection of viral DNA in the plasma, the development of an antibody response, and the presence of viral transcripts in the lymph nodes.

Furthermore, this model has been used to analyze coinfection with HHV-6A and simian immunodeficiency virus (SIV), a simian counterpart of HIV-1 typically used for experimental infection in macaques (Lusso et al., 2007). This approach provided the first *in vivo *data showing that HHV-6A infection can accelerate AIDS progression. Indeed, although HHV-6A infection did not seem to have any effect on SIV spreading, dually infected animals exhibited faster depletion in CD4^+^ T cells than the singly SIV-infected ones. Interestingly, HHV-6A infection also resulted in a faster decrease in CD8^+^ T cell count, which could be due to HHV-6A-induced *de novo* expression of CD4 in these cells.

A potential mechanism explaining the enhancement of AIDS by HHV-6A was suggested following the analysis of the virus isolated from monkeys receiving either single SIV infection or HHV-6A/SIV coinfection (Biancotto et al., 2009).* In vitro* replication of viral isolates obtained from singly infected monkeys was inhibited by coinfection with HHV-6A, and treatment with the chemokine CCL5 (regulated upon activation normal T cell expressed and secreted, RANTES) had similar effects. In contrast, all isolates from dually infected monkeys appeared to be resistant to both CCL5 treatment and HHV-6A infection, suggesting that *in vivo* coinfection with HHV-6A probably directs SIV evolution toward CCL5 resistance. Thus HHV-6A infection could create a high-CCL5 environment *in vivo*, in which CCL5-resistance would be advantageous for efficient SIV replication.

#### The marmoset model: evidence for HHV-6A-induced neurological disease

A recent study described a new model of infection by both HHV-6A and -6B using common marmosets (*Callithrix jacchus*), which represents so far the only model of HHV-6A infection associated with the more important clinical signs (Leibovitch et al., 2013). Indeed, HHV-6A-infected monkeys that received several monthly i.v. injections developed clear neurological symptoms, including motor weakness and sensory abnormalities. Moreover, magnetic resonance imaging (MRI) analyses revealed the presence of hyperintense lesions in the brain of one animal. This study provided the first *in vivo* evidence that HHV-6A infection is able to trigger neurological disease. In humans, both HHV-6A and -6B DNA was shown to be present in the brain of healthy people, indicating that both species have similar neuroinvasive properties. In marmosets, viral DNA was also occasionally detected in the brain of HHV-6A- and -6B-infected animals, which confirmed the capacity of both viruses to reach the brain. However, while HHV-6A infection led to evident neurological symptoms, infection with HHV-6B remained asymptomatic, thus showing an important difference between HHV-6A and -6B in their ability to infect marmosets (**Table 1**).

Interestingly, an additional group of marmosets was infected with HHV-6A through the intranasal (i.n.) route of inoculation, which resulted in radically different clinical outcomes. Based on histological data, the i.n. pathway was proposed as a possible route of transmission and access to the brain for HHV-6A in humans (Harberts et al., 2011). Contrary to i.v.-injected marmosets, i.n.-injected animals did not exhibit any sign of disease. Moreover, i.n.-inoculated animals rarely developed antibody responses and maintained plasma viremia, whereas i.v. injection led to the development of HHV-6-specific antibody responses and clearance of viral DNA in the plasma. These results suggested that the neurological symptoms observed with i.v. injection might be due to the immune response developed against the virus rather than to the direct consequences of viral infection and spreading. This model therefore emphasizes the importance of the route of inoculation in viral pathogenesis, and provides a clear *in vivo* demonstration that HHV-6A can cause a neurological disease with MS-like symptoms. The marmoset model thus appears as an appropriate model for the analysis of HHV-6A-induced neurological disease, and confirms the correlation between HHV-6 infection and the development of multiple sclerosis.

## MURINE MODELS

The possibility of using a murine model to study HHV-6 infection has been attractive to the scientific community since the discovery of HHV-6. However, mice were initially described to be resistant to HHV-6 infection (Lusso, 1996). Nevertheless, a few studies have described the use of *in vitro* or *in vivo* murine models for the study of HHV-6.

### SUSCEPTIBILITY OF MURINE CELLS TO HHV-6 INFECTION

The susceptibility to infection by HHV-6A and HHV-6B of several human and non-human cell lines was analyzed in a few studies. Both viruses failed to replicate in the murine mammary carcinoma cell line FM3A and viral transcripts were not detected in these cells, suggesting that murine cells are not permissive to infection by HHV-6 (De Bolle et al., 2005a). However, another study showed that infection by HHV-6A and HHV-6B enables the transcription of viral genes in murine primary oligodendrocyte precursors, although viral replication was not observed (Mock et al., 2006), suggesting that the susceptibility to HHV-6 infection may, to some extent, depend on the cell type analyzed. In addition, both HHV-6A and HHV-6B could induce cell cycle arrest in these cells, similarly, to what was previously observed in human oligodendrocyte precursor cells (Dietrich et al., 2004), indicating that some murine cell types could be used as a model to study certain aspects of HHV-6 infection *in vitro*.

### *IN VIVO* MURINE MODELS FOR THE STUDY OF HHV-6 INFECTION

It has been reported that natural resistance of mice to herpesvirus infection, particularly against herpes simplex virus (HSV) is genetically determined and linked to major histocompatibility complex (MHC) genes (Lopez, 1975). Balb/c mice were among the most susceptible lines and were recently used to analyze the link between HHV-6B infection and allergy (Svensson et al., 2010). Although systemic infection was not observed, inoculation of UV-inactivated virus resulted in the development of specific IgG responses and had protective effects against the development of allergy by limiting the inflammation in lungs, thus suggesting the immunosuppressive effects of HHV-6B *in vivo*.

To overcome natural resistance of mice to HHV-6 infection another approach using immunodeficient mice was developed, aiming to provide an *in vivo* environment for the study of human tissue rather than to analyze the infection in mice. For this purpose, severe combined immunodeficiency (SCID) mice were used. These mice carry a mutation which provokes profound T and B lymphopenia, allowing the engraftment of heterologous tissues (McCune, 1996). SCID mice were humanized by coimplanting human fetal thymus and liver under the murine kidney capsule, permitting the growth of a unique thy/liv organ which histologically resembles human thymus. Mice carrying thy/liv organ support human lymphopoiesis, thus allowing the study of human lymphoid cells in an *in vivo* context, and were used for the study of other human viruses, especially for human immunodeficiency virus (HIV; Van Duyne et al., 2009). Inoculation with HHV-6A or -6B was performed by direct injection in the implant and led to productive infection of human thymic cells, associated with a strong thymic depletion (Gobbi et al., 1999). That study demonstrated that HHV-6 infection is able to induce immunosuppression in an *in vivo *context, which may explain how HHV-6 could enhance the progression of immunodeficiency in AIDS patients. In this model, HHV-6 seemed to exhibit a particular tropism toward intra-thymic T progenitor cells (ITTPs), a rapidly dividing subset of thymic cells which gives rise to other thymocytes at later stages of maturation. This study suggested that lytic infection of ITTPs may play an important role in the HHV-6-induced thymic depletion.

Coinfection with HHV-6A or-6B and HIV-1 was later performed using the same model (Gobbi et al., 2000). Both viruses were found to be able to simultaneously infect the engrafted human tissue, yet infection with either virus did not seem to have any impact on the replication or virulence of the other.

### TOWARD NOVEL TRANSGENIC MURINE MODELS

Other models of humanized mice are currently under investigation. A model of rag2^-/- ^γc^-/-^ mice, deficient for T and B lymphocytes and NK cells and engrafted with human hematopoietic stem cells (Chicha et al., 2005) is being developed for the analysis of HHV-6 (Tanner et al., 8th International Conference on HHV-6&7, April 2013). The use of this model has allowed numerous advances in the field of retrovirology (Van Duyne et al., 2009) and will certainly help in the understanding of HHV-6 immunopathogenesis.

**FIGURE 1:**
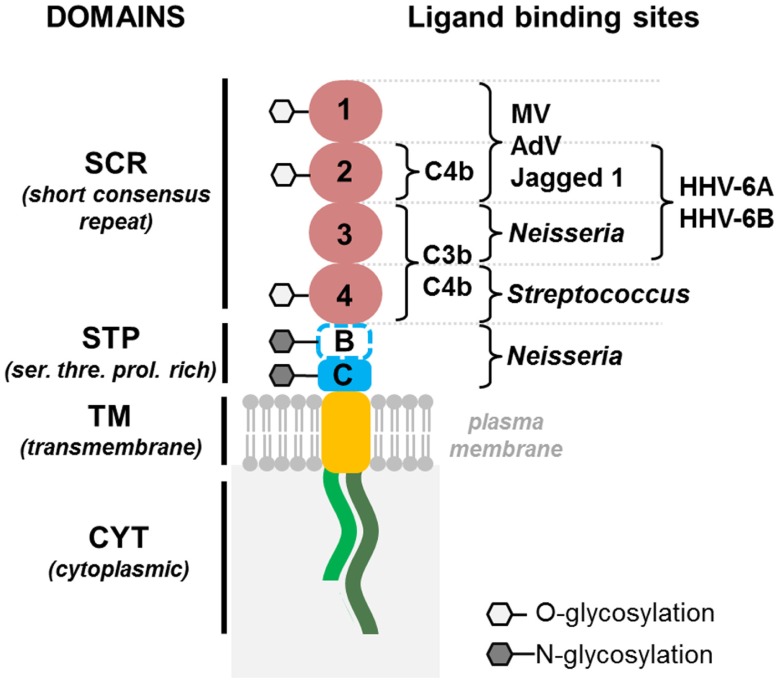
**Schematic representation of human HHV-6 receptor CD46, expressed in transgenic mice**. CD46 consists of an extracellular part, with indicated binding site for different known CD46 ligands, transmembrane domain and one of two cytoplasmic tails: short, Cyt-1 or long, Cyt-2. MV, Measles Virus; AdV, Adenovirus.

Human herpesvirus-6 was shown to use the human protein CD46 as a cellular receptor for viral entry (Santoro et al., 1999). This transmembrane protein is involved in the protection of host cells against complement lysis (Liszewski et al., 1991), through binding to C3b and C4b components of the complement (**Figure 1**), and was identified as the receptor for a variety of pathogens, including measles virus (vaccine strains), several serotypes of adenovirus and some pathogenic bacteria (Riley-Vargas et al., 2004). Moreover, it was recently found to bind the immunoregulatory molecule Jagged 1, a member of the Notch system (Le Friec et al., 2012; **Figure 1**). CD46 is ubiquitously expressed in humans and is mostly conserved in other primates (Seya et al., 1998). As viral tropism is determined by the pattern of expression of virus-specific cellular receptors, these molecules are key players in viral infection. Contrary to most primate CD46 proteins, murine CD46 has a lower percentage of identity with the human protein and its expression is restricted to the testis, which may account for the resistance of mice to infection. Therefore the generation of transgenic mice expressing human CD46 with a ubiquitous distribution, as in humans, could provide new perspectives for the development of animal models for HHV-6 infection. We have produced several lines of CD46 transgenic mice (Horvat et al., 1996; Marie et al., 2002) and used them to analyze the pathogenesis of HHV-6 infection. HHV-6A seemed to establish long-term persistence in the brain of these mice, and to induce leukocyte infiltration (Reynaud et al., 8th International Conference on HHV-6&7, April 2013). Thus, CD46 transgenic mice may represent a potential new small animal model for the study of HHV-6A-induced neuroinflammation. Some studies have suggested that CD46 may not be the only receptor for HHV-6B entry (Mori et al., 2002, 2004), opening thus, perspectives for the development of additional transgenic models for this virus. The availability of many experimental tools for murine models should facilitate further studies of virus–host interaction and HHV-6 pathogenesis.

## CONCLUSION

The development of relevant animal models is critical for a better understanding of viral pathogenesis, generating new diagnostic tools and assessing antiviral therapeutics and vaccines. Although animal models usually do not mimic all the aspects of the human disease, they do reproduce at least some of them and could thus help in a better understanding of certain aspects of viral pathogenesis. The number of animal models to study HHV-6 infection is still rather limited and mainly includes non-human primates. Utilization of pig-tailed macaques provided evidence for the HHV-6-induced acceleration of AIDS and recently HHV-6A infection in marmosets has strongly suggested a link with neurological diseases. The latent nature of HHV-6 infection makes most *in vivo* studies often difficult to carry out. Recent advances in the development of murine models for HHV-6 infection, with numerous and powerful tools available, should be of critical help for in-depth immunobiological and genetic studies of HHV-6 infection.

## Conflict of Interest Statement

The authors declare that the research was conducted in the absence of any commercial or financial relationships that could be construed as a potential conflict of interest.
